# Automated Detection of Neurodevelopmental Disorders Using Face-to-Face Mobile Technology Among Typically Developing Greek Children: Randomized Controlled Trial

**DOI:** 10.2196/53465

**Published:** 2024-10-11

**Authors:** Eugenia I Toki, Victoria Zakopoulou, Giorgos Tatsis, Jenny Pange

**Affiliations:** 1 Department of Speech and Language Therapy School of Health Sciences University of Ioannina Ioannina Greece; 2 Laboratory of New Technologies and Distance Learning Department of Early Childhood Education University of Ioannina Ioannina Greece

**Keywords:** main principles, automated detection, neurodevelopmental disorders, principal component analysis, early screening, early intervention, detection, screening, assessment, digital tool, serious game, child, Greece, speech, psychomotor, cognitive, psychoemotional, hearing, machine learning, apps, predictions, screening, prognosis

## Abstract

**Background:**

Neurodevelopmental disorders (NDs) are characterized by heterogeneity, complexity, and interactions among multiple domains with long-lasting effects in adulthood. Early and accurate identification of children at risk for NDs is crucial for timely intervention, yet many cases remain undiagnosed, leading to missed opportunities for effective interventions. Digital tools can help clinicians assist and identify NDs. The concept of using serious games to enhance health care has gained attention among a growing group of scientists, entrepreneurs, and clinicians.

**Objective:**

This study aims to explore the core principles of automated mobile detection of NDs in typically developing Greek children, using a serious game developed within the SmartSpeech project, designed to evaluate multiple developmental domains through principal component analysis (PCA).

**Methods:**

A total of 229 typically developing children aged 4 to 12 years participated in the study. The recruitment process involved open calls through public and private health and educational institutions across Greece. Parents were thoroughly informed about the study’s objectives and procedures, and written consent was obtained. Children engaged under the clinician’s face-to-face supervision with the serious game “Apsou,” which assesses 18 developmental domains, including speech, language, psychomotor, cognitive, psychoemotional, and hearing abilities. Data from the children’s interactions were analyzed using PCA to identify key components and underlying principles of ND detection.

**Results:**

A sample of 229 typically developing preschoolers and early school-aged children played the Apsou mobile serious game for automated detection of NDs. Performing a PCA, the findings identified 5 main components accounting for about 80% of the data variability that potentially have significant prognostic implications for a safe diagnosis of NDs. Varimax rotation explained 61.44% of the total variance. The results underscore key theoretical principles crucial for the automated detection of NDs. These principles encompass communication skills, speech and language development, vocal processing, cognitive skills and sensory functions, and visual-spatial skills. These components align with the theoretical principles of child development and provide a robust framework for automated ND detection.

**Conclusions:**

The study highlights the feasibility and effectiveness of using serious games for early ND detection in children. The identified principal components offer valuable insights into critical developmental domains, paving the way for the development of advanced machine learning applications to support highly accurate predictions and classifications for automated screening, diagnosis, prognosis, or intervention planning in ND clinical decision-making. Future research should focus on validating these findings across diverse populations integrating additional features such as biometric data and longitudinal tracking to enhance the accuracy and reliability of automated detection systems.

**Trial Registration:**

ClinicalTrials.gov NCT06633874; https://clinicaltrials.gov/study/NCT06633874

**International Registered Report Identifier (IRRID):**

RR2-https://doi.org/10.3390/signals4020021

## Introduction

Child development refers to a child’s growth, in speech, language, hearing, psychomotor, cognitive, and psychoemotional domains [1].

Speech and language development involves verbal and nonverbal communication skills [1]. Speech development refers to a child’s capacity to speak and communicate, starting from infancy with cooing and babbling and leading to single words, phrases, and sentences as the infant matures. It includes voice, articulation, and fluency [1,2]. Language development involves receptive and expressive language processes and includes vocabulary, grammar, syntax, interpreting complicated sentences, and social language use for communication [3,4].

Infancy begins with primary gestures and sounds and progresses to complicated language skills. Hearing underpins speech and language. Early hearing detection with regular hearing screenings [5] contributes toward the early identification and treatment of hearing abnormalities supporting children to communicate with others [6,7].

Psychomotor development refers to gross motor skills, fine motor skills, and visual-motor integration [1]. Thoroughly, gross motor skills involve using and coordinating vast muscle groups to crawl, walk, jump, run, and climb stairs and help children move and play [8]. Fine motor skills involve the coordination of small muscle groups, particularly those in the hands and fingers. Tasks such as picking up small objects, holding a pencil, cutting with scissors, and tying shoelaces require fine motor abilities. Visual-motor integration refers to vision and movement coordination, enabling children to replicate shapes, write, and accomplish various hand-eye coordination activities [1].

Cognitive development is a child’s capacity to problem-solve, think, reason, and understand the environment [9,10]. Genetics, environment, education, and explorative experiences impact cognitive development. Critical thinking includes memory (recalling information), attention (focusing and ignoring distractions), problem-solving, and abstract thinking (the ability to think beyond tangible experiences and grasp abstract notions).

Socioemotional development involves emotional development, social development, self-concept, self-esteem, and emotional intelligence [11]. Emotional development entails not only the awareness and expression of one’s feelings but also the ability to regulate those feelings in an appropriate manner. Emotional development is an important factor affecting children’s well-being and interpersonal relationships [12]. Social development is an aspect of a child’s development focusing on the ability to interact with other people and the capacity to create and sustain connections. Empathy, sharing, taking turns, and a grasp of social standards are all important components of a social life [13]. Self-concept and self-esteem refer to how children think about themselves, their capabilities, and their overall value [11].

Overall, the many aspects of a child’s development are intertwined, and advancement in one area often facilitates development in others. Both the importance of developing gross motor skills as well as cognitive development for school readiness and long-term academic success has been recognized [14], and motor and cognitive developments, along with social and emotional developments, form the 3 main interrelated developmental areas in the early years of learning and development [8]. In an Australian toddler sample, levels of gross motor skills are positively associated with cognitive development [8]. Additional evidence of a close relationship between fine motor abilities and intelligence in children, both with and without a diagnosis of attention-deficit/hyperactivity disorder (ADHD), has been reported. Nevertheless, children diagnosed with ADHD exhibit stronger relationships [15]. Essentially, it is crucial to a child’s healthy growth, learning, and overall well-being to have a comprehensive understanding of their development and to provide help in these areas. Genetics, environment, and individual strengths and obstacles also impact a child’s domain advancement. Tracking and encouraging development in all domains promotes holistic growth and success in school, relationships, and well-being.

The clinical manifestation of abnormal development exhibits notable variations among newborns, toddlers, and teenagers and the impact on young children can be significant, leading to a range of health conditions with detrimental effects on their personal, academic, social, and occupational functioning [16].

**Figure 1:**
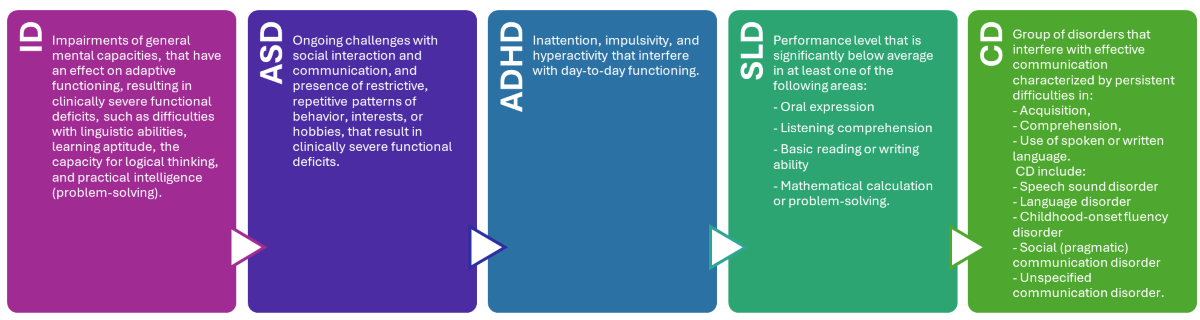
DSM-5 visualization of included NDs profile patterns. ADHD: attention-deficit/hyperactivity disorder; ASD: autism spectrum disorder; CD: communication disorder; DSM-5: Diagnostic and Statistical Manual of Mental Disorders (Fifth Edition); ID: intellectual disability; ND: neurodevelopmental disorder; SLD: specific learning disorder.

Children and adolescents exhibiting deficits in the developmental domains and significantly impacting their quality of life commonly face a disorder [17]. Indeed, neurodevelopmental disorders (NDs) are a set of conditions that often manifest in childhood characterized by deficits in brain development that impact numerous facets of an individual’s capacity to function, including communication, learning, social interaction, behavior, cognitive processes, and emotional well-being [18-20]. The clinical features of various NDs are used to describe the disorders presented in the *DSM-5* (*Diagnostic and Statistical Manual of Mental Disorders* [Fifth Edition]) [21]. *DSM-5* defines NDs including intellectual disability, autism spectrum disorders (ASDs), ADHD, specific learning disorder (SLD), and communication disorders [21]. The *DSM-5* assigns certain features to the profiles of these disorders, including exaggerations, weaknesses, and delays in reaching developmental milestones [21,22]. A summary of the profile features of included NDs in *DSM-5* is shown in Figure 1, which visualizes their patterns.

It is essential to distinguish between normal development and disorders that emerge in the early stages of development to understand NDs. This statement refers to the different aspects of human development, including genetic, neural, cognitive, and socioemotional domains [23]. As NDs form a considerable heterogeneous group with a broad spectrum of clinical features characterized by convergent boundaries and common co-occurrence [24], differentiation among NDS is also required. In addition, the early emerging and continuing course of NDs emphasizes the need for early diagnosis and intervention [25].

Developmental tests are required to predict the phenotype of children’s delays in achieving developmental milestones, as well as to monitor their later progress [21]. They obtain information from observation in multiple domains [20], clinical history, and standardized assessments [26-29].

Moreover, assessment processing can be advantageous in distinguishing between these disorders due to their high prevalence and significant interindividual variability [30]. It is crucial to thoroughly test chosen measures for their construct reliability and validity [31]. Besides, new and flexible diagnostic procedures would lead the clinician to consider the type of ND to design proper policies and educational practices, thus facilitating the implementation of well-specialized and individualized comprehensive interventions [23,32].

It is well documented that games play an important role in child development [33-36]. Serious games (SGs) are games created with a primary goal other than pure entertainment [37]. SGs are used in many sectors such as education, training, health care, sustainability projects, marketing purposes, scientific investigation, crisis management, environmental planning, engineering, politics, arts, and more.

SGs have been used in various health issues, encompassing chronic diseases, mental health disorders, rehabilitation with physical activity, substance abuse/management, obesity and eating disorders, geriatric care and cognitive impairment, chronic pain management, and asthma management [38]. In the present era, there has been a notable increase in the use of artificial intelligence (AI) [39-41]. Consequently, integrating AI with SG aims to provide a personalized and engaging health care experience. Diverse AI algorithms have been used to address a range of health concerns through the use of SG, with motor impairment as the most targeted one [42]. Furthermore, SG commonly uses AI for various purposes including rehabilitation, diagnosis, personalized screening, performance assessment, monitoring, risk modeling, drug discovery, and therapy response prediction. The main purposes of AI in an SG have been noted to be (1) disease or disorder detection and (2) user-performance evaluation [42].

Neurodevelopmental difficulties can be monitored through SG, which can be used for screening, early detection, diagnostic assessment, objective measurements, engagement, motivation, and personalized therapy [22,42-44]. Empowered Brain (Brain Power), a wearable technology combining smart glasses with AI-powered software and an augmented reality game, was found to improve social communication and attention in teenagers with ASD and ADHD [45]. A new iPad tool using the TrueDepth camera (Apple, Inc) tracks emotions and attention to diagnose ASD and ADHD, showing 78% and 45% accuracies in tests on adults and children. However, additional research is needed to enhance accuracy [46]. The ADHD360 project aims to create a platform for the early detection and management of ADHD in children interacting with a serious game, “Pizza on Time” using advanced machine learning algorithms. Preliminary results indicate that the system can predict ADHD in participants aged 7-16 years with up to 85% accuracy [47]. Galexia (Pambú! Developers) is a game-based tool designed to diagnose dyslexia in children aged 6-12 years. It is a cost-effective and nonintrusive alternative for large-scale screening, with future work aiming to develop a comprehensive tool for specific learning difficulties [48]. Kim et al [49] used convolutional neural network analysis to explore the correlation between developmental disabilities and motor skills in DoBrain mobile games, revealing significant differences [49].

Diagnostic tests and objective measurements are standardized and repeatable [50] and digital health tools can improve diagnosis time [51]. Additionally, SG can motivate children with neurodevelopmental problems to engage in pleasant upgraded and anxiety-free procedures. These studies collectively highlight the significant advancements and the growing potential of SG and digital diagnostics in ND detection. They underscore the need for continued research to improve accuracy, expand diagnostic capabilities, and ensure these tools are accessible and effective across diverse populations and settings toward individualized, efficient, and effective clinical experiences in communication, cognition, emotion, and behavior.

In this study, we are exploring the key elements of automated detection based on the earlier theoretical developmental domains using a new SG. We conducted a principal component analysis (PCA) on typically developed Greek children as part of this process.

## Methods

### Overview

This section describes the study’s ethical considerations, the study design, the study sample, the SG and activities, data collection sources and formulation, and the analytical procedure.

This study is part of an ongoing research project titled “Smart Computing Models, Sensors, and Early Diagnostic Speech and Language Deficiencies Indicators in Child Communication,” with the acronym “SmartSpeech” funded by the Region of Epirus and supported by European Regional Development Fund.

### Ethical Considerations

The study was conducted in accordance with the Declaration of Helsinki and approved by the Research Ethics Committee of the University of Ioannina, Greece (protocol code 18435, approved on May 15, 2020). The project’s nature, purpose, and procedures were thoroughly explained to parents during an informative meeting, ensuring compliance with the General Data Protection Regulation (GDPR). All participating parents gave informed written consent after being provided with detailed information regarding the study’s objectives, procedures, and ethical guidelines. The informed consent process ensured that parents understood the study’s compliance with GDPR. To protect participants’ privacy, the data collected in this study were deidentified. All necessary measures were taken to ensure the confidentiality of participant information throughout the study. No compensation was provided to participants. Care was taken to ensure that no individual participants or users could be identified in any images included in the paper (or its supplementary materials).

### Study Design

The research was conducted in educational and health care settings across Greece. As this is a randomized trial, the CONSORT (Consolidated Standards of Reporting Trials) 2010 checklist was used to report information included in this study. Participants were recruited through an open call distributed via private and public health and educational establishments across the country. The recruitment focused on parents of typically developing children aged 4 to 12 years. Parents were invited to informational meetings where the study’s objectives, procedures, and ethical guidelines were explained. This session covered the study’s purpose, the nature of the SG “Apsou” by SmartSpeech Project, and the expected involvement of their children. Parents received comprehensive information about the study during these sessions and were asked to provide informed written consent for their children’s participation to ensure compliance with GDPR and ethical considerations. The study included typically developing preschool and school-aged children without NDs, other medical conditions, or medications that could affect the results. Under clinicians’ face-to-face supervision (2021-2022), children played the SG “Apsou,” designed to measure various developmental domains, including speech, language, psychomotor, cognitive, psychoemotional, and hearing abilities [43]. This structured approach ensured comprehensive and ethical recruitment, engaging parents and children while adhering to ethical guidelines and data protection standards.

The study included 229 preschool and school-age children, 118 (51.5%) of whom were boys, and 111 (48.5%) were girls. The mean age of the children was 6.88 (SD 1.94) years. All the children in the sample were attending typical educational settings. We state that all participants included in the sample completed the whole SG.

The SG Apsou, version 1.0.1, developed within the SmartSpeech project, is designed to detect children at risk of NDs. It offers a web-based platform with various activities targeting different developmental skills, making the assessment process engaging and effective. The game is designed around a narrative plot, allowing players to use existing skills or acquire new ones in a scenario focusing on motivation, narrative, and fun [52]. The digital gamified environment uses video game mechanics to turn evaluation into a game, leveraging the capabilities of Unity (Unity Technologies), a cross-platform game engine, for smooth gameplay with high-quality graphics and animations (Multimedia Appendix 1) [53,54]. The game is designed for mobile devices (Android, iOS, Windows) and collects data from parents, clinicians, and the child’s gameplay, providing a comprehensive data set for analysis [53,54]. The validation process for the Apsou game involves several steps to ensure its effectiveness in assessing NDs, including data analysis using various AI algorithms. The game’s effectiveness is evaluated by comparing the performance of typically developing children and children diagnosed with NDs. The integer-bound neural network outperforms other methods, paving the way for future research to improve the game’s detection effectiveness [22,43].

To assess neurodevelopmental skills, an interdisciplinary team designed this SG based on theories that measure speech and language, psychomotor, cognitive, psychoemotional, and hearing skills. There are 18 main variables in total, and each domain is measured by the child’s performance on specific tasks within the SG activities. The SG software assesses NDs in various domains, and Table 1 (“Apsou” SG variables used to assess NDs Domains) describes the variables used to assess NDs domains.

**Table 1:** “Apsou” SG^a^ variables used to assess ND^b^ domains.

Domains of assessment	SG ND variables of assessment
Speech and language development	Verbal and intellectual ability Verbalization after instruction Targeted voicing activities Articulation Phonology Syntax Pragmatic perception
Psychomotor development	Fine motor skills Prewriting skills Spatial orientation Sequencing
Cognitive development	Memory Recognition Perception or discrimination Sustained attention Cognitive flexibility
Psychoemotional development	Empathy
Hearing	Conditioned play audiometry

^a^SG: serious game.

^b^ND: neurodevelopmental disorder.

Upon completion, the child’s responses are processed and evaluated using a scoring system [43]. The system collects and examines the scores in the 18 variables of the game assessment to create a developmental individualized profile for the child. All variable scores are computed on a scale of 0 to 100.

The SG was developed to collect children’s responses during playtime [53,55], represented as variables that fall into one of these input sources categories: (1) hand moving on the touch screen (ie, forming sequencing procedures, sentence formation, or reordering objects); (2) verbal responding to questions or executing instructions (ie, verbalization after initiation, word recognition [speech to text], used in articulation, pragmatics perception); and (3) clicking buttons and objects or drag and dropping on screen (ie, pictures with animals and shapes)

The SG automatically generated scoring performance for touch screen hand movement responses according to accuracy. The rest of the variables were analyzed as follows.

SmartSpeech uses speech-to-text (STT) to recognize a child’s verbal responses during the SG. Participants were asked to pronounce characters’ names and objects, with predetermined correct answers. CMUSphinx (Carnegie Mellon University), a free, fast, and offline speech-to-text program, was used [56]. A Greek recognition model was constructed and trained in the software [57], detecting the best word to match the child’s input, to determine a correct answer. The software detected the word best matches what the child had said and, consequently, whether the child gave a correct answer or not. The recognized speech responses by the STT software are evaluated to the predetermined set of words that constitute a correct answer and thus turning into scores by assigning 0 to false and 1 to true.

The system is also designed to connect and collect biometric data from the child, such as heart rate and eye-tracking metrics, but these are outside the scope of this study [22,43].

Descriptive statistics were used to analyze typically developing children’s gaming performance. *z* scores as standardized scores were calculated to provide information about the distance and direction of each observation from the mean value for that specific variable. We examine our variables with a reliability test analysis via the Cronbach α coefficient, which is used to evaluate the internal consistency of our measurements. To reduce the number of original features present in the data set while retaining crucial information, the widely recognized PCA method for dimensionality reduction was used [58]. PCA was used to examine the data structure and determine the main principles for automated detecting NDs. For the analysis following, we used the IBM SPSS Statistics (version 28).

## Results

**Figure 2:**
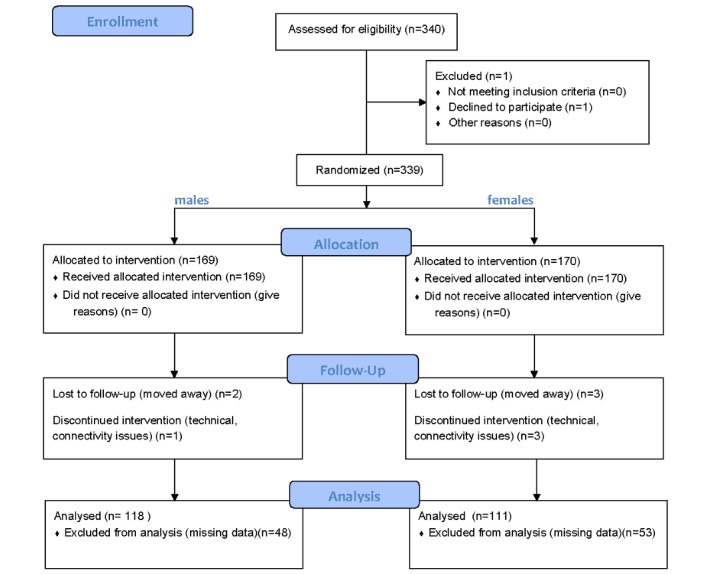
The study’s CONSORT flow diagram.

In this study, 229 children (males: n=118, 51.5%; females: n=111, 48.5%) with a mean age of 6.88 (SD 1.94) years (2021-2022) completed the SG different activities and tasks that are meant to assess specific neurodevelopmental issues as described in Figure 2, presenting the study’s CONSORT flow diagram. These tasks are grouped into 18 variables, each related to a different skill. The mean scores and SD of the children’s performance in the 18 variables are listed in Table 2.

**Table 2:** Mean scores and SD of the 229 typically developing Greek children’s performance in the 18 variables of the “Apsou” game.

Variable	Values, mean (SD)
Verbal and intellectual ability	31.00 (10.46)
Verbalization after Instruction	15.28 (36.06^a^)
Targeted voicing activities	35.07 (25.19)
Articulation	14.57 (16.08^a^)
Phonology	81.12 (24.17)
Syntax	37.69 (37.16)
Pragmatic perception	83.29 (18.26)
Fine motor skills	70.74 (28.54)
Prewriting skills	31.27 (18.51)
Spatial orientation	36.62 (11.38)
Sequencing	60.61 (15.77)
Memory	23.29 (21.28)
Recognition	26.64 (9.21)
Perception or discrimination	38.43 (14.10)
Sustained attention	28.40 (8.83)
Cognitive flexibility	63.39 (18.74)
Empathy	38.25 (13.13)
Conditioned play audiometry	26.64 (26.05)

^a^The variables are not normally distributed, which can result in an SD greater than the mean due to skewness.

The data set was standardized, and *z* scores of all the above variables were computed to take values with a mean of 0 (SD 1). This coefficient takes values between 0 and 1, with the higher value resulting in a higher degree of reliability.

For our data set, the Cronbach α coefficient was 0.805, a relatively high value according to the literature [59]. The internal consistency of the data, calculated using the SPSS, was found to be satisfactory. As part of the procedure, the α coefficient was calculated iteratively by removing a variable from the set each time. The coefficients showed slight deviations, but the variable “prewriting skills” had the greatest impact. If this variable is removed from the data set, the α coefficient for the remaining 17 variables is calculated as 0.810. In order to identify the key components in our data, we used PCA to retain as much of the original information as possible. To ensure that PCA was suitable for analyzing the study’s data, we made the following assumptions: the Kaiser-Meyer-Olkin measure of sampling adequacy was calculated at 0.738, greater than 0.70, signifying that the current data was appropriate for applying PCA. Furthermore, the Bartlett test of sphericity (*P*<.001) was presented in Table 3, rejecting the null hypothesis that the correlations between the variables were all 0. Both tests indicated that this study’s data was suitable for applying PCA.

**Table 3:** Kaiser-Meyer-Olkin measure of sampling adequacy and Bartlett test for data set’s suitability for PCA^a^ on 18 variables measured in the SG^b^ “Apsou” among 229 typically developing Greek children.

PCA			Values
Kaiser-Meyer-Olkin measure of sampling adequacy			.738
**Bartlett test of sphericity**			
	Approximate chi-square (*df*)	1637.912 (153)	
	*P* value	<.001	

^a^PCA: principal component analysis.

^b^SG: serious game.

We first examined the extraction process of the initial components and focused on the initial eigenvalues to understand the significant components. Table 4 shows the commonalities of the variables in our analysis and explains the proportion of each variable’s variance. The values closer to 1 are considered better. “Fine motor skills” (0.863) was the most crucial variable in our observation, while “verbalization after instruction” (0.235) was the least important one.

**Table 4:** Communalities of the variables in the PCA^a^.

Variable	Initial	Extraction
Verbal and intellectual ability	1.000	0.853
Verbalization after instruction	1.000	0.235
Targeted voicing activities	1.000	0.857
Articulation	1.000	0.786
Phonology	1.000	0.523
Syntax	1.000	0.618
Pragmatic perception	1.000	0.517
Fine motor skills	1.000	0.863
Prewriting skills	1.000	0.637
Spatial orientation	1.000	0.798
Sequencing	1.000	0.499
Memory	1.000	0.472
Recognition	1.000	0.340
Perception or discrimination	1.000	0.481
Sustained attention	1.000	0.850
Cognitive flexibility	1.000	0.731
Empathy	1.000	0.639
Conditioned play audiometry	1.000	0.361

^a^PCA: principal component analysis.

**Figure 3:**
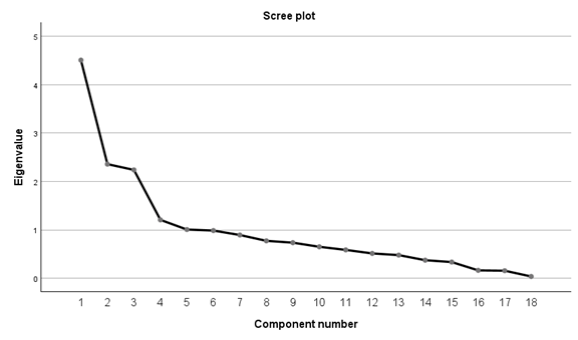
Eigenvalues of 5 main PCA in a scree plot of NDs measured by the SG “Apsou.” ND: neurodevelopmental disorder; PCA: principal component analysis; SG: serious game.

We used the eigenvalue-1 criterion to determine the number of “meaningful” components for this PCA. In Table 5, it is shown that the 5 principal components (PCs) account for a cumulative variance of 61.44% of the initial variance. The 5 PCs with eigenvalues being greater than 1, which was confirmed using the scree plot (Figure 3). Specifically, the PCA revealed new features represented by components PC1-PC5. PC1 accounts for the highest variance (24.914%), followed by PC2 (12.513%), PC3 (11.713%), PC4 (6.657%), and PC5 (5.643%). The first 3 principal components, PC1, PC2, and PC3, explain a total cumulative variance of 49.139% of the initial variance, whereas the other 2 (PC4 and PC5) with an eigenvalue greater than 1 explained a total cumulative variance of 12.301% of the initial variance. In other words, the components explained a total cumulative variance of 61.440% of the initial variance, and as Figure 3 shows, components PC1-PC5 cannot explain all the variability. Thus, we applied a varimax rotation.

**Table 5:** Total variance explained by principal components.

Component	Initial eigenvalues				Rotation sums of squared loadings		
	Total	Percentage of variance	Cumulative percentage	Total		Percentage of variance	Cumulative percentage
1	4.484	24.914	24.914	4.484		24.914	24.914
2	2.252	12.513	37.427	2.252		12.513	37.427
3	2.108	11.713	49.139	2.108		11.713	49.139
4	1.198	6.657	55.797	1.198		6.657	55.797
5	1.016	5.643	61.440	1.016		5.643	61.440
6	0.975	5.416	66.857	N/A^a^		N/A	N/A
7	0.831	4.616	71.473	N/A		N/A	N/A
8	0.811	4.504	75.977	N/A		N/A	N/A
9	0.736	4.086	80.064	N/A		N/A	N/A
10	0.692	3.842	83.906	N/A		N/A	N/A
11	0.620	3.442	87.348	N/A		N/A	N/A
12	0.544	3.020	90.368	N/A		N/A	N/A
13	0.485	2.695	93.063	N/A		N/A	N/A
14	0.440	2.447	95.510	N/A		N/A	N/A
15	0.362	2.011	97.521	N/A		N/A	N/A
16	0.190	1.054	98.575	N/A		N/A	N/A
17	0.176	0.977	99.551	N/A		N/A	N/A
18	0.081	0.449	100	N/A		N/A	N/A

^a^N/A: data are not applicable or not available.

As such, we used a rotated component matrix using varimax with Kaiser normalization. By doing this, we determined the “loads” placed on the various original features for each of the 5 PCs. Table 6 illustrates the rotated component matrix that reports the stronger correlations between the PCs and the initial variables. The correlation coefficients with absolute values lower than 0.4 are hidden for better readability.

**Table 6:** Rotated component matrix using varimax rotation.

Variable	Component				
	1	2	3	4	5
Pragmatic perception	0.704	—^a^	—	—	—
Syntax	0.669	—	—	—	—
Sequencing	0.665	—	—	—	—
Phonology	0.615	—	—	—	—
Memory	0.609	—	—	—	—
Perception or discrimination	0.518	—	—	—	0.458
Recognition	0.494	—	—	—	—
Verbal and intellectual ability	—	0.899	—	—	—
Articulation	—	0.877	—	—	—
Empathy	—	0.746	—	—	—
Targeted voicing activities	—	—	0.925	—	—
Spatial orientation	—	—	0.882	—	—
Cognitive flexibility	—	—	0.806	—	—
Sustained attention	—	—	—	0.893	—
Fine motor skills	—	—	—	0.820	—
Conditioned play audiometry	—	—	—	0.586	—
Prewriting skills	—	—	—	—	0.784

^a^Not applicable.

For each PC, the most critical original features are as follows.

- PC1: Pragmatic perception, syntax, sequencing, phonology, memory, perception or discrimination, and recognition
- PC2: Verbal and intellectual ability, articulation, and empathy
- PC3: Targeted voicing activities, spatial orientation, and cognitive flexibility
- PC4: Sustained attention, fine motor skills, conditioned play audiometry
- PC5: Prewriting skills and perception or discrimination

All original variables exhibit correlations with their respective PCs, indicating a strong association among them.

The current PCA indicates the main PCs, PC1-PC5, suggesting accordingly communication skills, speech and language development, vocal processing, cognitive skills and sensory functions, and visual spatial skills.

## Discussion

### Principal Findings

This study conducted data analysis on typically developed preschoolers and first school-aged children, exploring the main principles toward automated detection of NDs and the underlying theoretical principles. The age range of 4-12 years was selected for this study because NDs often become apparent during the early school years and persist into adulthood. It is important to identify these disorders early for timely intervention. During this age range, children reach important developmental milestones in speech, language, cognition, and motor skills. By examining this age group, we can gain insight into standard developmental paths and the natural variations within them, and we can carry out a thorough assessment during a crucial stage of growth. This ensures that the “Apsou” serious game can efficiently recognize and assist children at a pivotal moment. This strengthens the significance and influence of the study on early childhood and primary education.

The PCA resulted in 5 PCs, as per the Kaiser rule. Only the PCs having eigenvalues greater than 1 were retained. These components describe a cumulative variance of 61.44%, which is due to the intraindividual differences in children’s performance across some domains [60].

As noted by Van Geert and Van Dijk [61], “intraindividual variability can be defined as differences in the level of a developmental variable within individuals and between repeated measurements.” Developmental change is often preceded by variability, which plays a crucial role in exploration and selection processes.

It has been observed that individuals exhibit a wide range of performances in different tasks, such as verbalization after instruction as shown in Table 2. These variations in performance can be attributed to individual skillfulness, level of readiness, and the acquisition of different skills and milestones. These findings emphasize the complexity of typical development, which is characterized by variations in child developmental rhythm, abilities, and performances at each stage. These results are consistent with other research findings [1,62,63].

It is widely acknowledged that the distinctions observed in child development indicate various developmental phases. Although children may attain developmental milestones at varying ages, the sequence in which they achieve these milestones can differ among children [60,64].

Besides, these results pinpoint findings on preschoolers and first school-aged children as this study establishes the fundamental developmental skills that support the automated digital system’s ability to classify the child’s performance per developmental stage. Relevant findings regarding developmental age have been reported [54]. Further, children may temporarily sample advanced milestones but may not fully master them until later and can jump between stages [60,64]. Thus, individuals’ actions are inherently unique, contributing to the observed variability in human behavior. Diversity is crucial for children's development as it manifests unique problem-solving approaches, highlighting interindividual diversity. The recorded total variation is expected to underline typical multifaceted heterogeneous profiles of children with NDs.

Furthermore, researchers from various scientific fields have shown a growing interest in early human development [65]. This growth in interest can be attributed to the recognition of the wide range of NDs, which are characterized by significant genetic and phenotypic differences. Additionally, there is a critical need to enhance our understanding of the similarities and differences among various syndromes, disorders, and disease processes. The primary focus of studying early human development and predicting neurodevelopmental outcomes is to examine critical aspects such as the presence and progression of distinct physical and neurological characteristics, as well as potential abnormalities in neurobehavioral and psychopathological patterns.

The results of the current study revealed 5 main new features (PC1-PC5). These PCs are logically aligned with the original features to establish their compatibility with the domains examined in the traditional manner of assessing NDs. According to the results of the PCA, the first 3 PCs explain about 50% of the total cumulative variance. Further, this study’s findings agree with the globally accepted theoretical principles [21] and are also in line with other research work as follows.

The first new feature (PC1) explaining the most variance in the PCA, includes original features (1) pragmatic perception, (2) syntax, (3) sequencing, (4) phonology, (5) memory, (6) perception or discrimination, and (7) recognition with the most important loads. These skills are pointing toward communication skills and are in line with the theoretical principles of NDs (intellectual disability, global developmental delay, communication disorders, ASD, ADHD, SLD, motor disorders, and other NDs) [1,3,21] that demonstrate deficiencies in “… mental functions in conceptual (language, reading, writing, mathematics, memory, insight, knowledge, interpretation), social (empathy, compassion, social judgment, communication and interaction skills, amicability, harmony) and practical (personal care, school and/or occupational responsibilities and organization, financial management, entertainment, hobby) aspects …” [66].

It has been found that the second new feature (PC2) is responsible for explaining the second highest variance in the PCA, which includes the original features of verbal and intellectual ability, articulation, and empathy, with the second most significant loads. PC2 indicates speech and language development and is in accordance with recent research underlying that observing a child’s speech and language development is crucial for their emotional well-being, especially in the early years [1,66]. Additionally, as children grow, their speech intelligibility improves over time. Typically, it starts at around 25% by the age of 2 and gradually gets better until they reach complete intelligibility by the age of 4. However, if a child is constantly struggling with their speech and language, such as using infantile language, mispronouncing words, or lacking spontaneous speech, it may indicate a delay in their speech and language development [67,68].

The third new feature (PC3) explains the third most variance in the PCA, including original features (1) targeted voicing activities, (2) spatial orientation, and (3) cognitive flexibility, with the subsequent most necessary loads. The interaction of these 3 original features indicates vocal processing, which is also described in accordance with another research. It is also considered a fundamental predictor of cognitive development along with language development [69]. For example, the observed modulation in pleasure vocalizations underscores an increasing association between the act of looking toward the caregiver (spatial-orientated emotional communication), while a gradual evolution undergoes because of the simultaneous presence of early spontaneous behaviors (vocal activities) and intentionally driven behaviors (cognitive flexibility) [70].

The fourth new feature (PC4) points to cognitive skills and sensory functions and explains the following most variance in the PCA, including original features: (1) sustained attention, (2) fine motor skills, and (3) conditioned play audiometry, with subsequent most important loads. In the same direction, current NDs commonly result in cognitive deficits such as attention and executive function problems [71]. For instance, measurements of visual or auditory sustained attention, vigilance, and impulsivity in children with ADHD often exhibit executive dysfunction-like behavior, which impairs their development. ADHD children have long-term neuropsychological deficits, including poor performance on visual or auditory vigilance tasks, response inhibition, attention, and goal-directed behavior [72]. In addition, children with developmental language impairments often display minor motor impairments [73] and reduced coding of spoken syllables and phonemes [74], which are frequently overlooked. However, identifying and treating nonlanguage impairments is advantageous to aid the children’s language development (ie, phonological processing) [75] preventing in the long run the development of cognitive deficits [76].

Lastly, the fifth new feature (PC5) suggesting visual-spatial skills, explains the next most variance in the PCA, including original features: (1) prewriting skills and (2) perception or discrimination with the later most important loads. In the same direction, it is well established that deficiencies in visual-spatial and visual-perceptual abilities, and difficulties solving problems early, associated with reading and attention problems, while language-based skills and IQ remain normal, are the hallmarks of nonverbal SLD [1]. Further, consistent with previous research, weaknesses in visual-spatial abilities, including prewriting skills, are reported in children at risk of SLD [77]. Moreover, consistent with other studies, a spectrum of visuospatial abilities (or visuospatial dimension), ranging from gifted to impaired abilities, exhibit early impairments in various areas, including visual-spatial attention [78], visual-constructive, spatial working memory and fine motor skills, and poor performances in educational subjects such as mathematics, as well as emotional and social problems [79].

Using a sample of typically developing children is fundamental to establishing a robust normative baseline against which deviations can be measured. By understanding the typical developmental trajectories and their inherent variability, atypical patterns that signify NDs can be more accurately pinpointed. This differentiation between normal developmental variations and clinically significant deviations ensures that automated detection methods are both sensitive and specific. It is interesting to note that the 5 PCA factors identified in this study were also found in a parallel relevant study [80] involving typically developing and ND children. Apsou SG, factor analysis, and machine learning with logistic regression were used to create a strong predictive model. The model achieved an area under curve of 0.730, accuracy of 0.815, *F*_1_-score of 0.776, precision of 0.823, and recall of 0.815, clearly indicating the predictive potential of these factors.

A shortcoming of this study is the absence of implementation of a diagnostic test of intelligence. To complement this limitation though, we took into consideration that (1) all the participants were attending the typical educational settings and (2) the Apsou clinical administrators confirmed the nonexistence of NDs in participants using the child's developmental and communicative background provided by the parent, and the observation approach applied during their interaction with the child during the game.

The accuracy of the transcription provided by STT software is a limiting factor in this research. We used the highly accurate CMUSphinx STT program [57], trained to convert adult verbal responses to text, but as the software improves over time, also the accuracy of models relying on these transcripts will improve as well. Further research may also use innovative other open sources for this purpose.

Although PCA is a justified method to reveal the most important features of NDs representing 61.44% of the variance, additional methods can be used to explore features validity like deep learning approaches, genetic and molecular data analysis, machine learning and statistical analysis, biological markers, neurophysiological and electrophysiological measures, nonlinear dimensionality reduction techniques, manifold learning, and more.

These findings have the potential to guide future research toward improving automated classification and prediction. This progress can play a crucial role in the timely and precise identification and treatment of NDs in clinical and educational settings. Understanding typical and atypical developmental patterns, including NDs, can significantly enhance clinical practice with advanced AI tools. Future research should combine these findings with cutting-edge AI and machine learning techniques to explore additional features such as biometric data and longitudinal tracking. This combined effort will not only enhance the accuracy and reliability of automated detection systems but also lead to more effective and personalized interventions. These developments will enable more tailored and holistic approaches to supporting individuals with NDs, ultimately improving their long-term outcomes and quality of life. Furthermore, future studies may strive to validate these findings across diverse populations, ensuring broader applicability and generalizability.

### Conclusions

In this study, the main principles for the automated detection of NDs were explored. A sample of 229 typical developed preschoolers and early school-aged children played a unique SG,” Apsou,” developed to measure developmental domains.

Using principal components analysis, we unveiled five core principles, signifying (1) communication skills, (2) speech and language development, (3) vocal processing, (4) cognitive skills and sensory functions, and (5) visual-spatial skills, thus corroborating the theoretical principles regarding typically developmental domains. These findings open the door for future research to harness them for further analysis in refining automated screening, diagnosis, prognosis, and treatment planning to support clinical decision-making on NDs.

## Appendix

Multimedia Appendix 1 Apsou version 1.0.1 screenshots.

Multimedia Appendix 2 CONSORT 2010 checklist.

## Abbreviations

ADHD: attention-deficit/hyperactivity disorder
AI: artificial intelligence
ASD: autism spectrum disorder
CONSORT: Consolidated Standards of Reporting Trials
DSM-5: Diagnostic and Statistical Manual of Mental Disorders (Fifth Edition)
GDPR: General Data Protection Regulation
ND: neurodevelopmental disorder
PC: principal component
PCA: principal component analysis
SG: serious game
SLD: specific learning disorder
STT: speech-to-text

## Data Availability

The data sets generated and analyzed during this study are not publicly available, as participants did not give written consent for their data to be shared publicly, but they are available from the corresponding author upon reasonable request.
