# Supplementary figures and images for: Automated Detection of Neurodevelopmental Disorders Using Face-to-Face Mobile Technology Among Typically Developing Greek Children: Randomized Controlled Trial
Source: JMIR Form Res. 2024 Oct 11;8:e53465. doi: 10.2196/53465 (PMC11512119; doi:10.2196/53465)

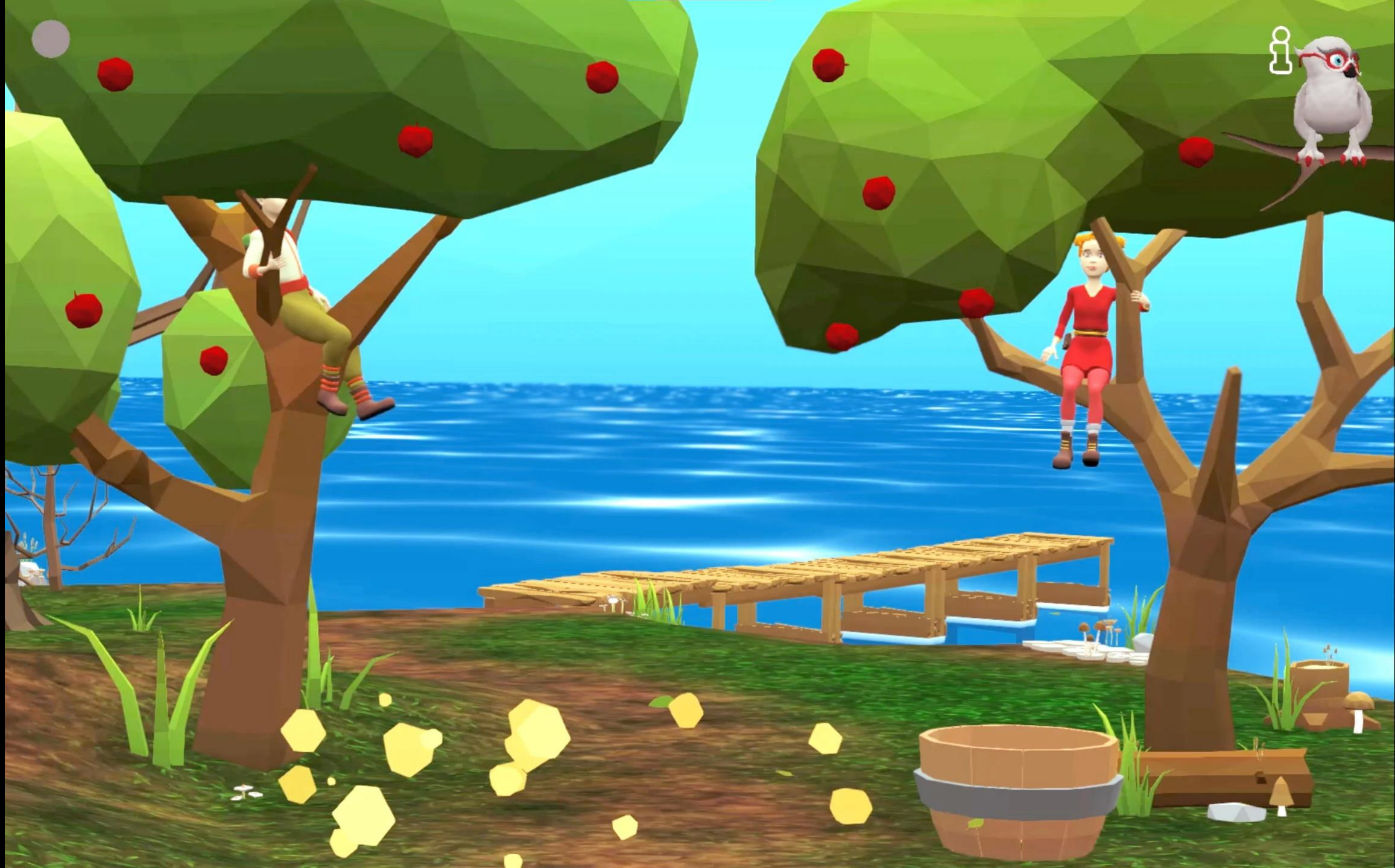

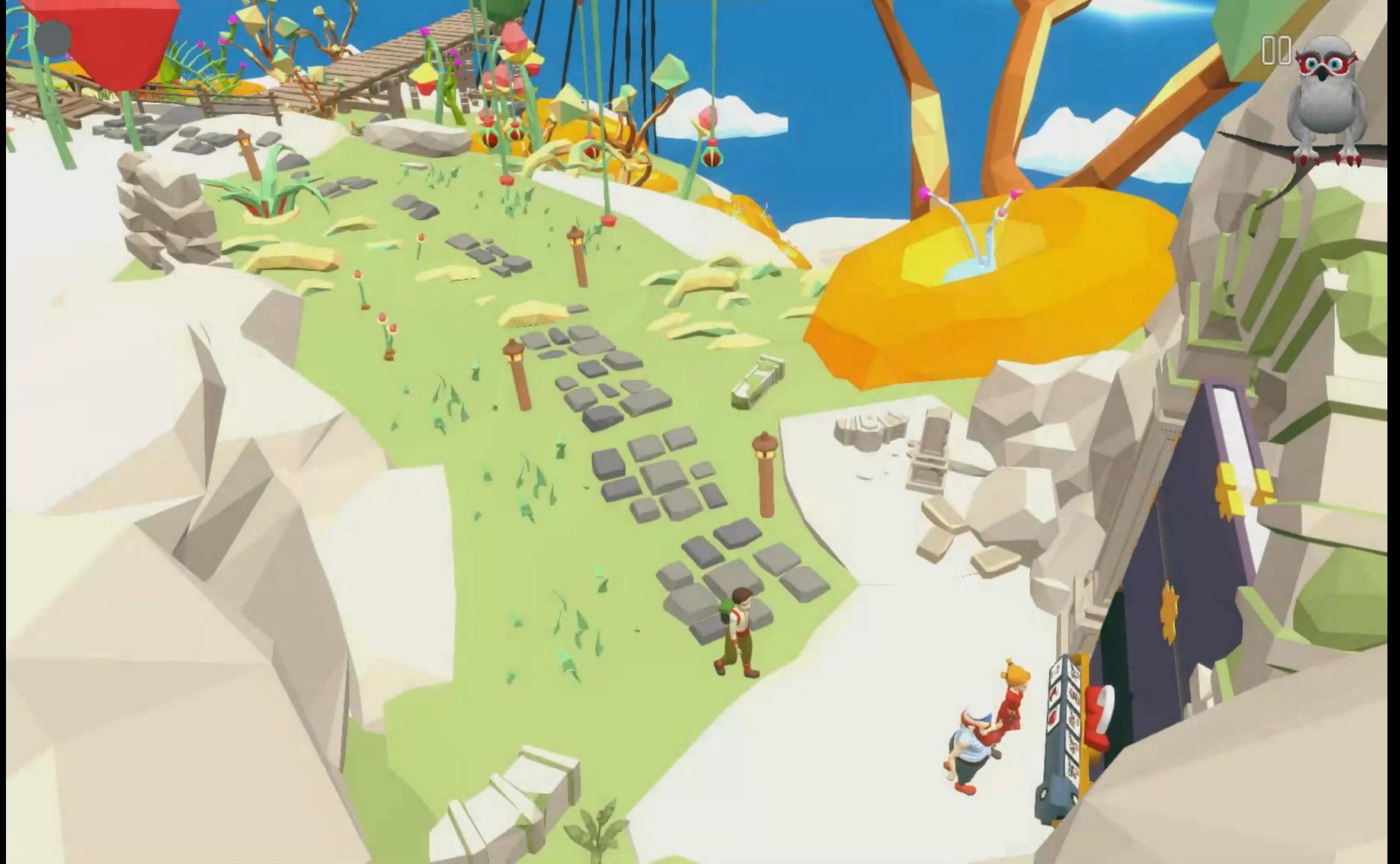

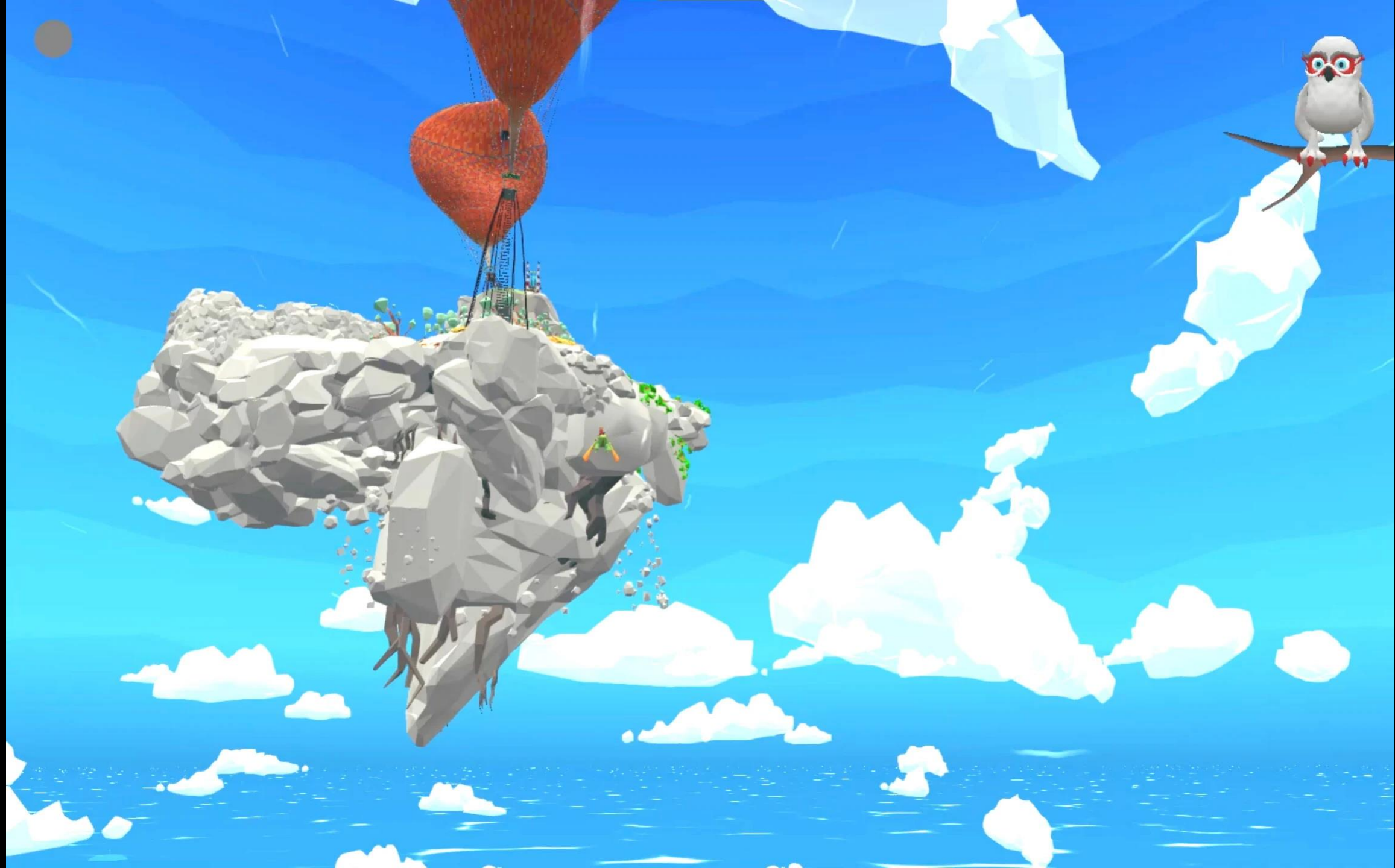

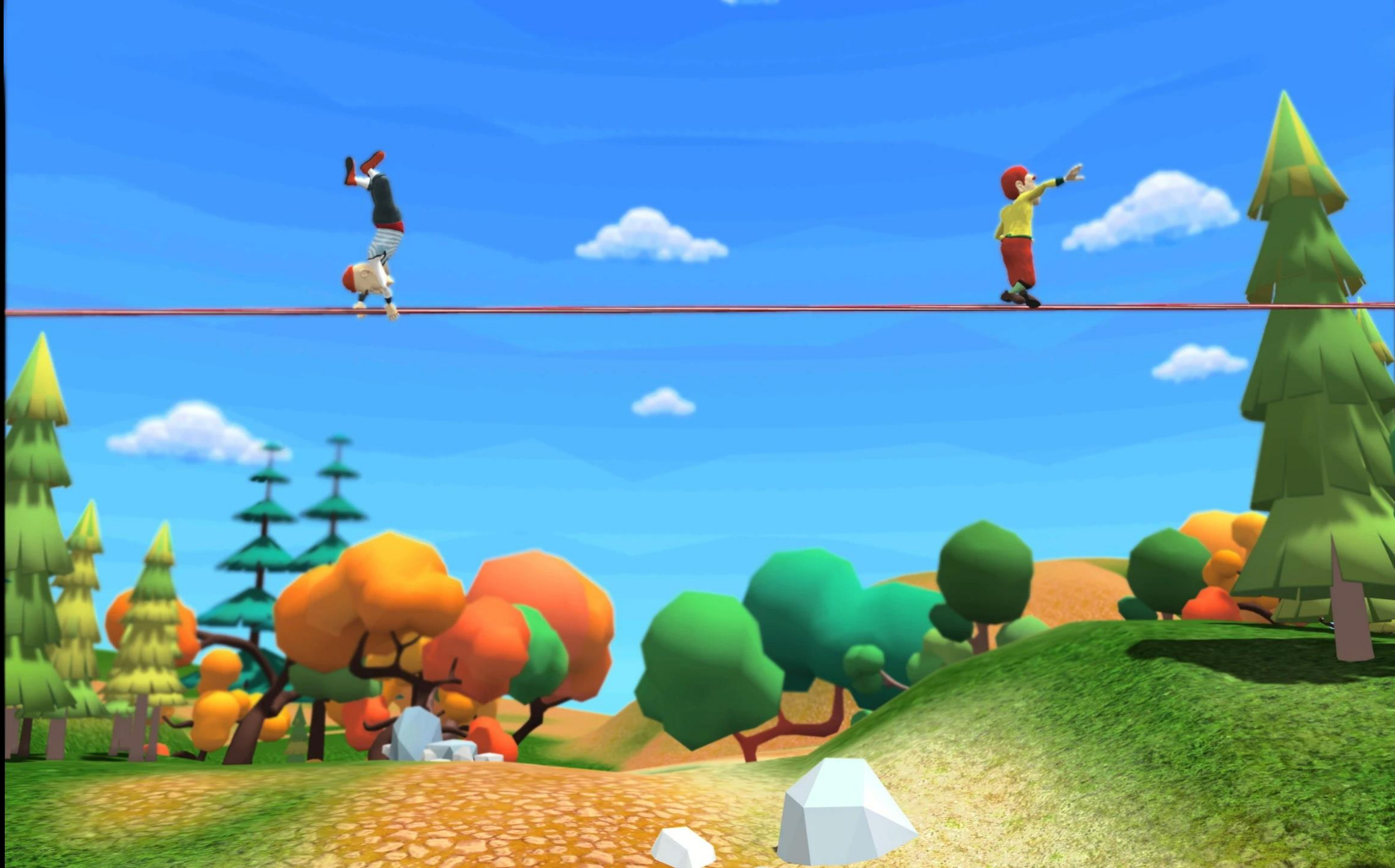

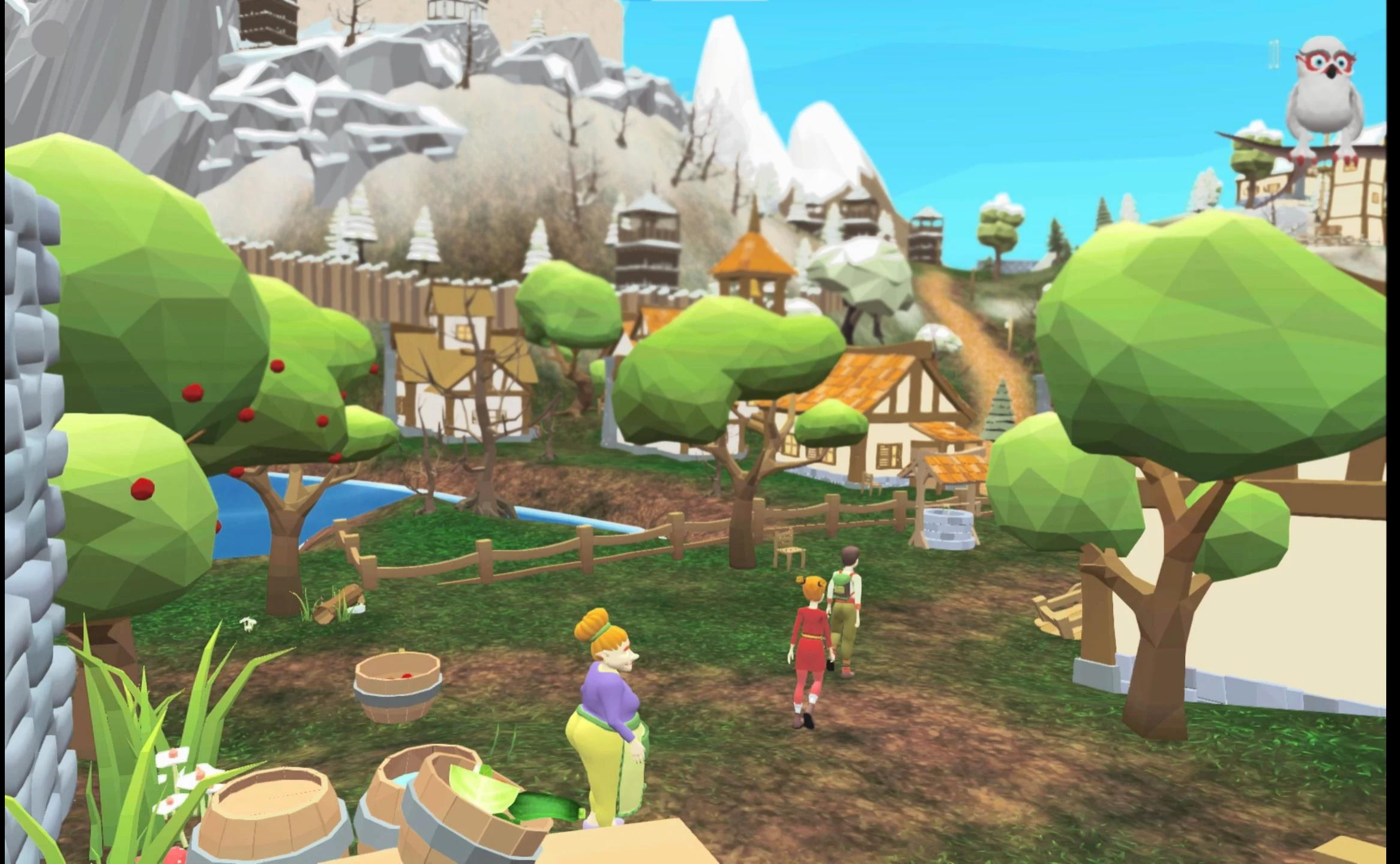

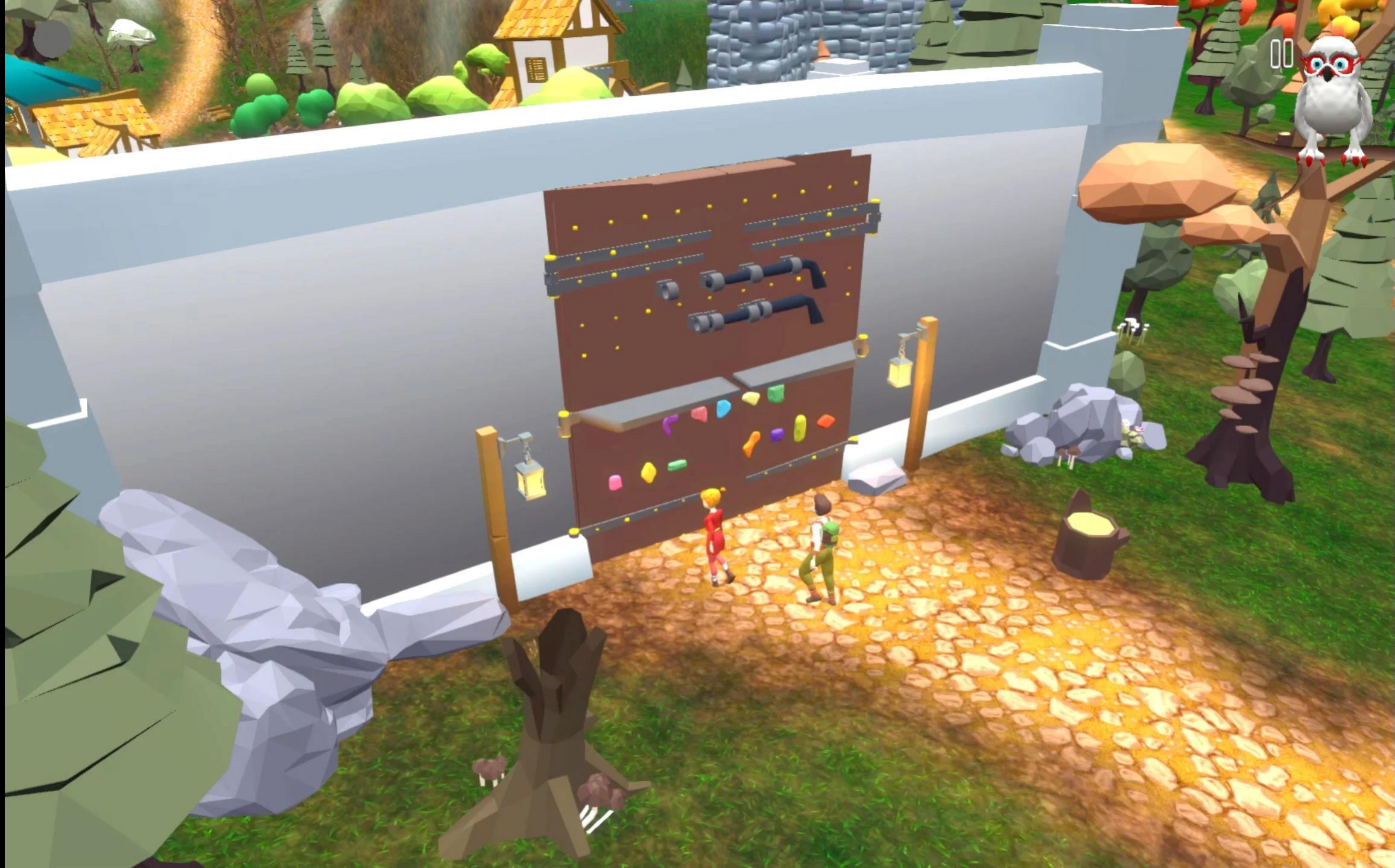

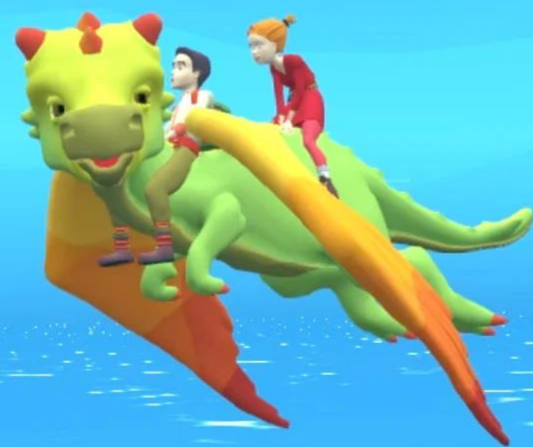

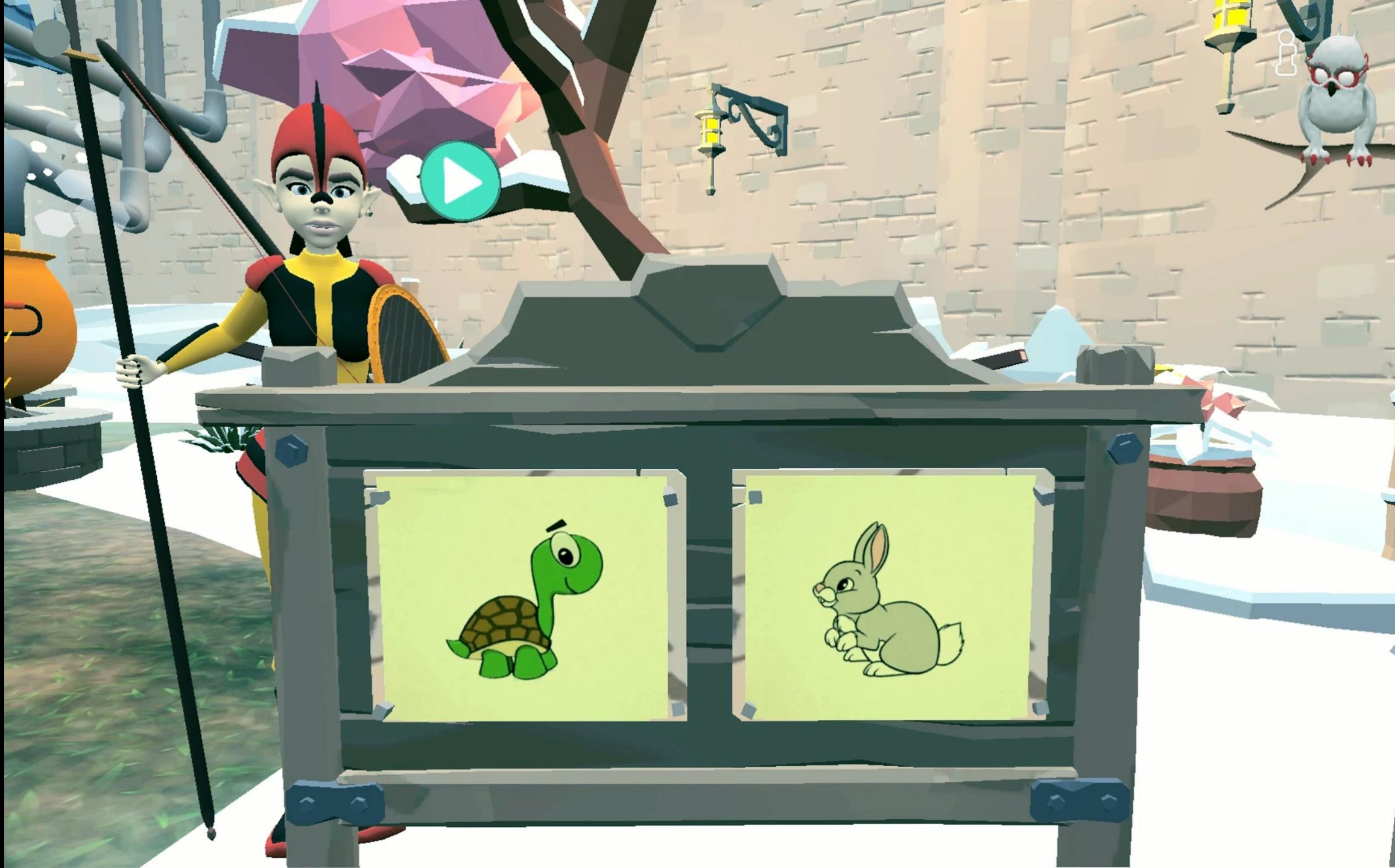

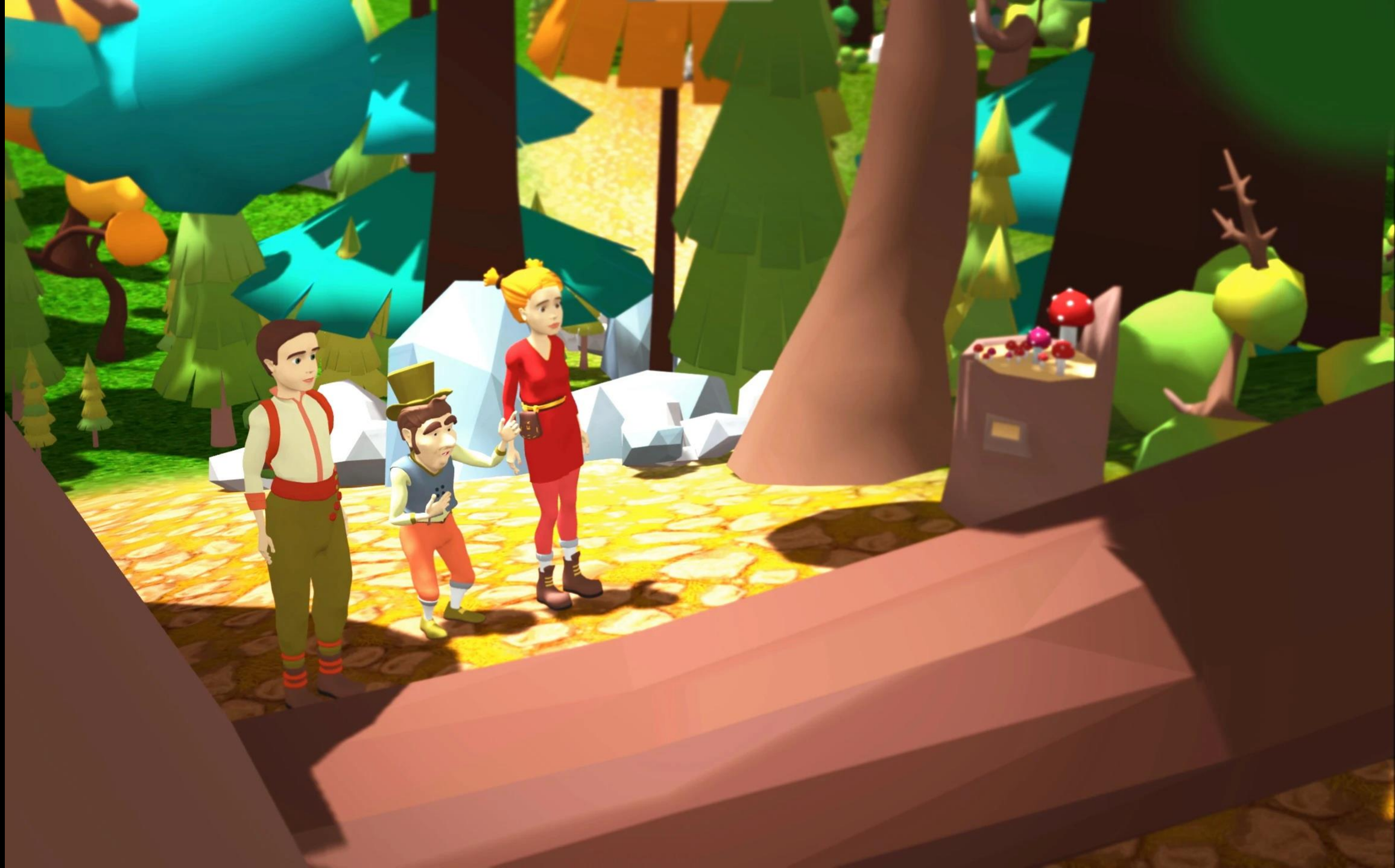

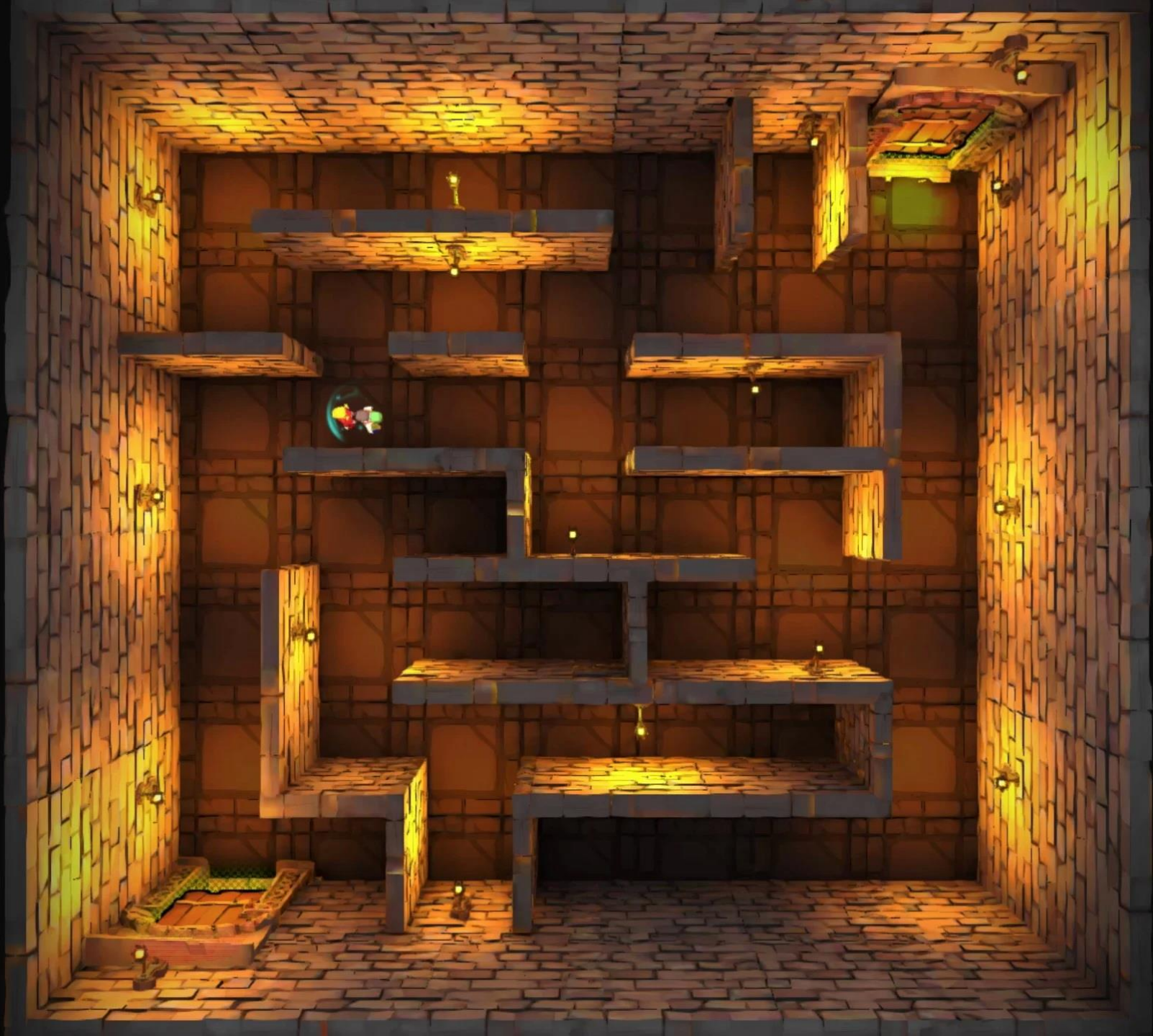

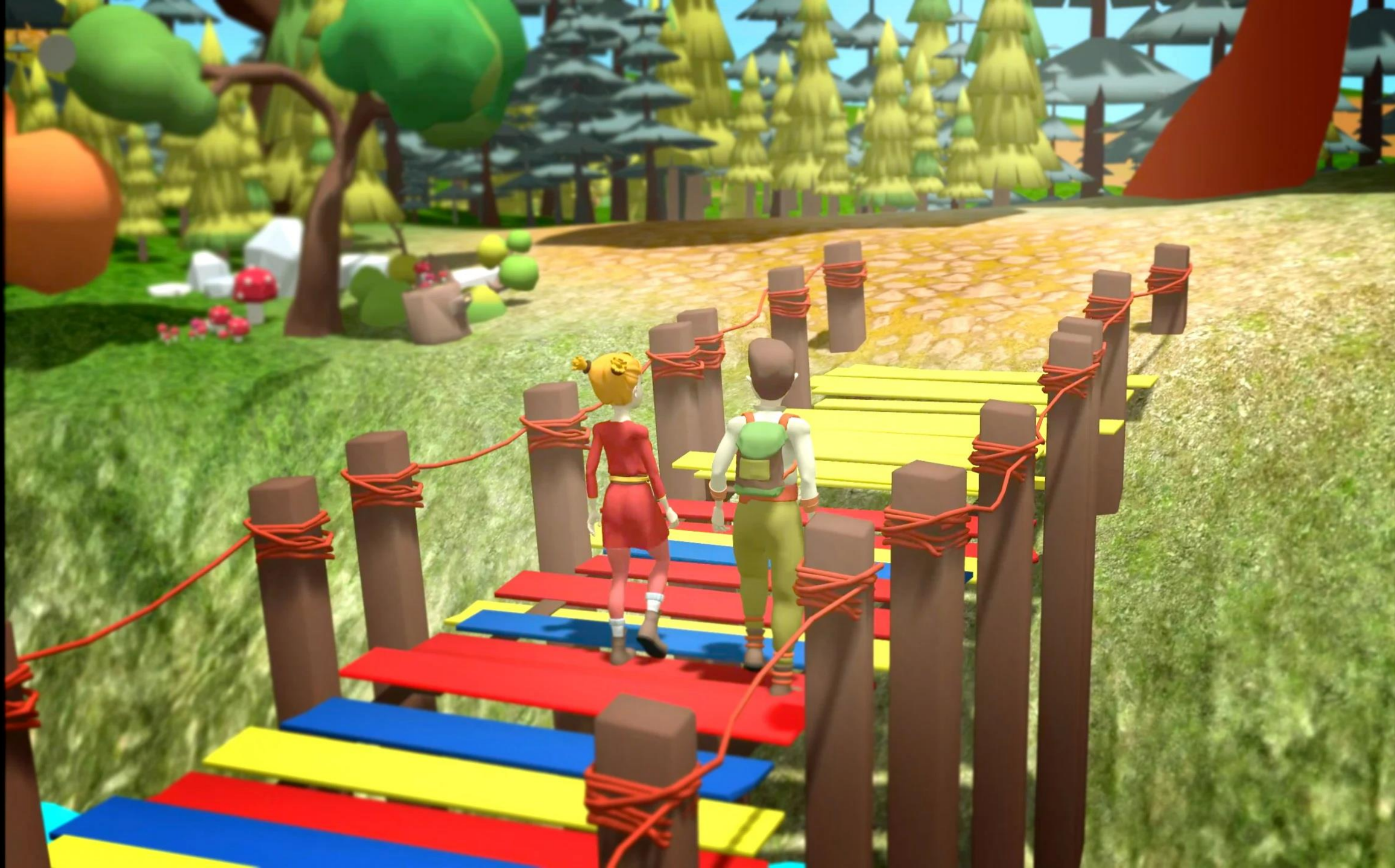

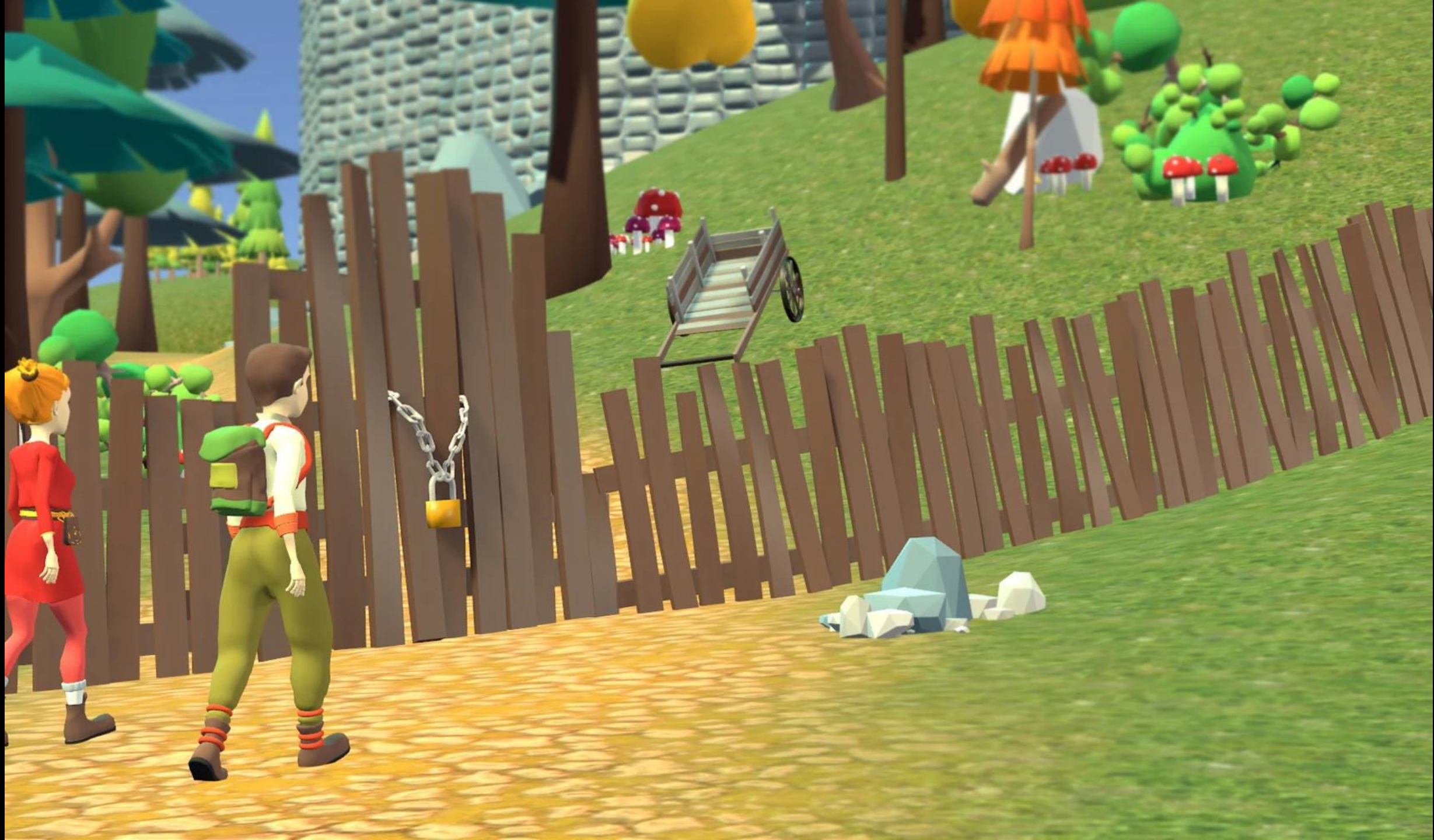

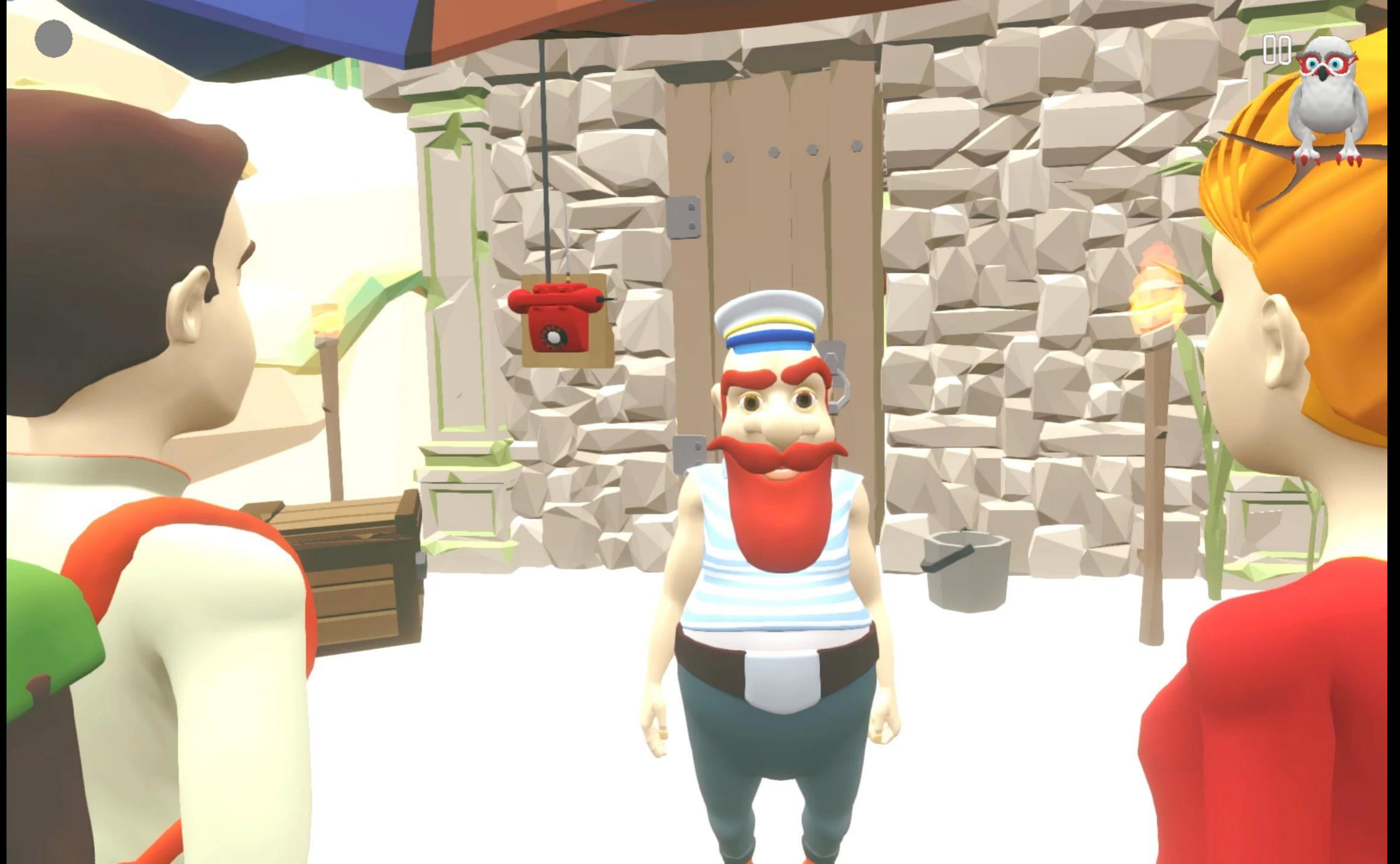

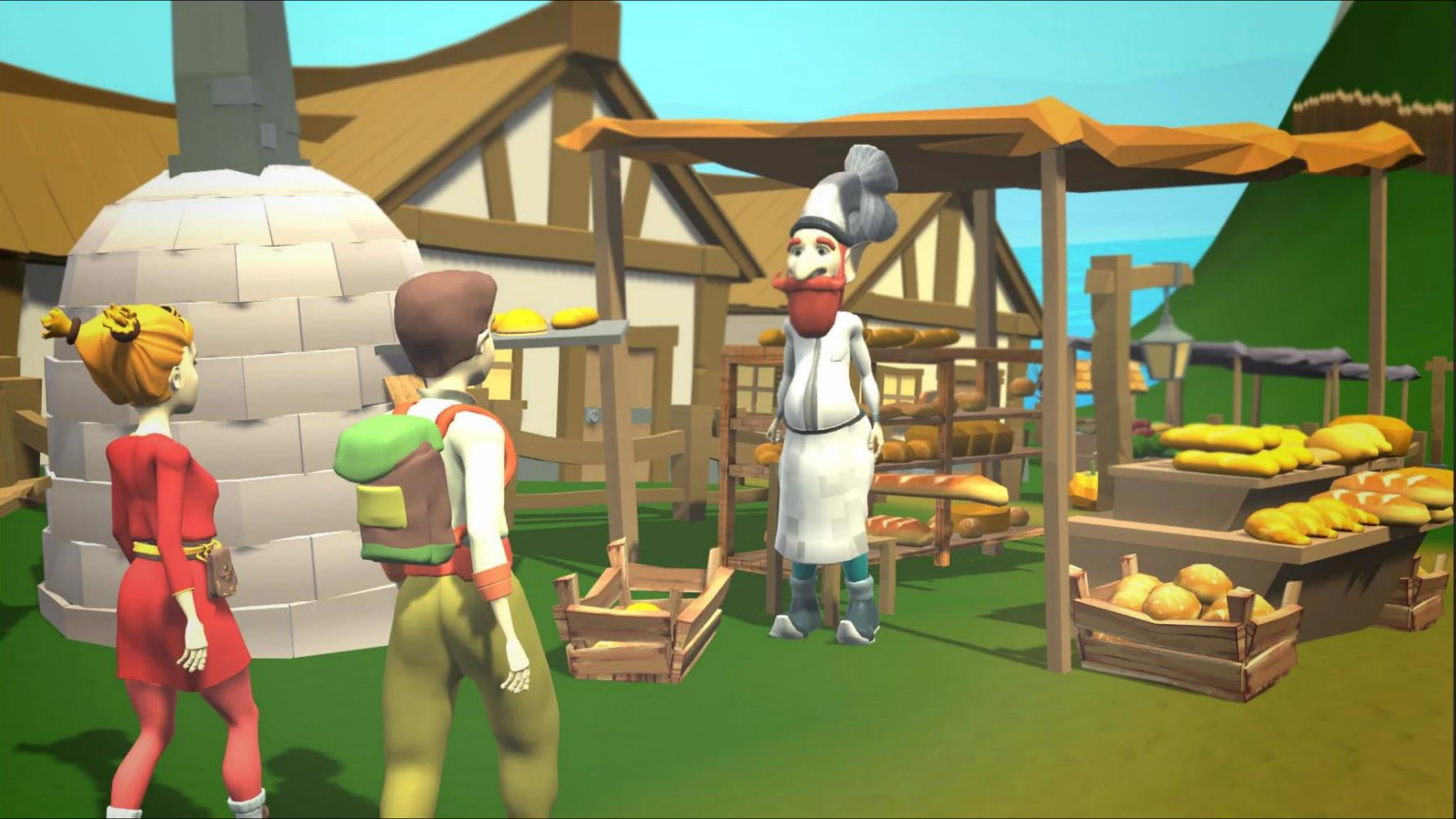

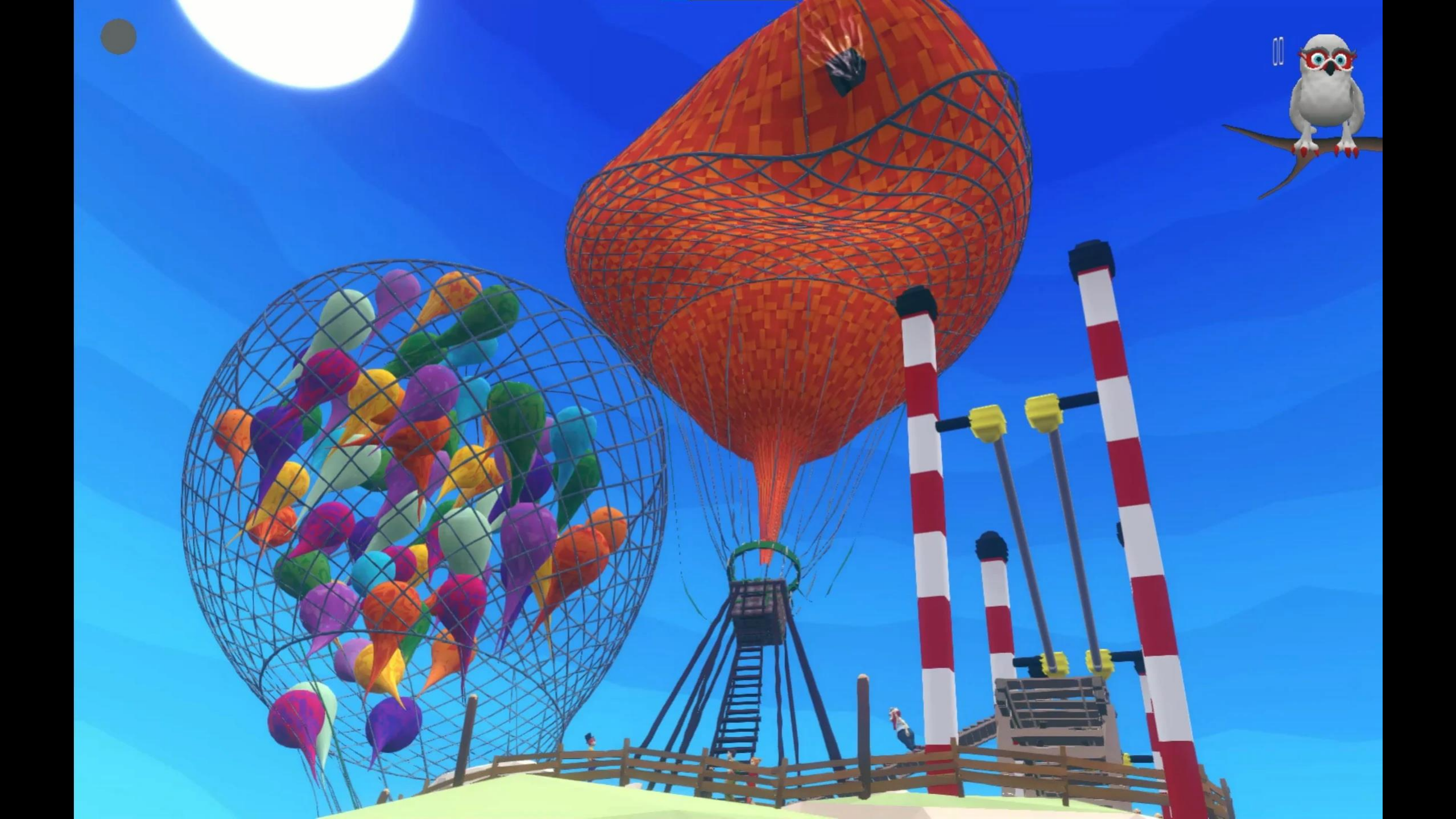

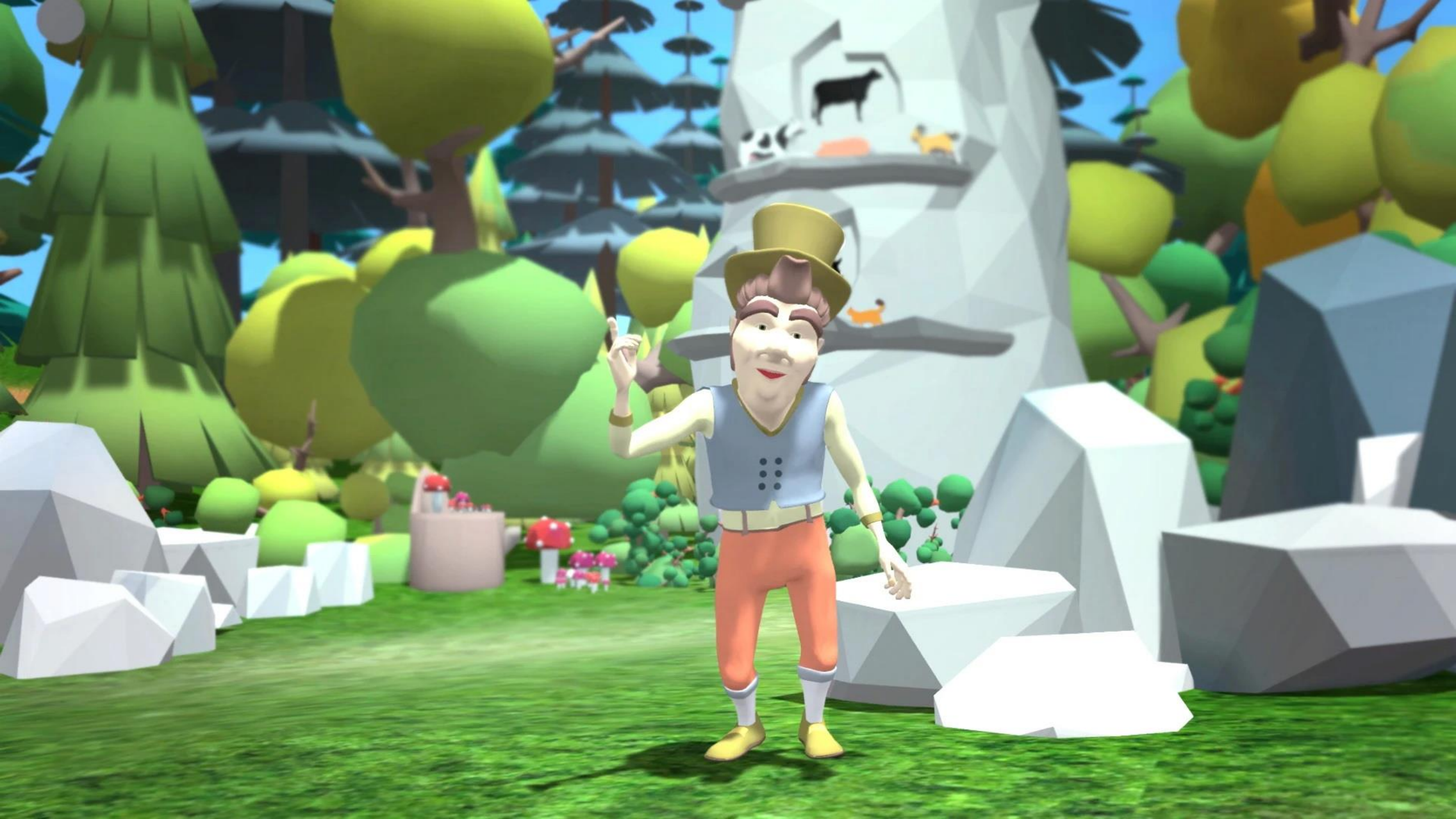

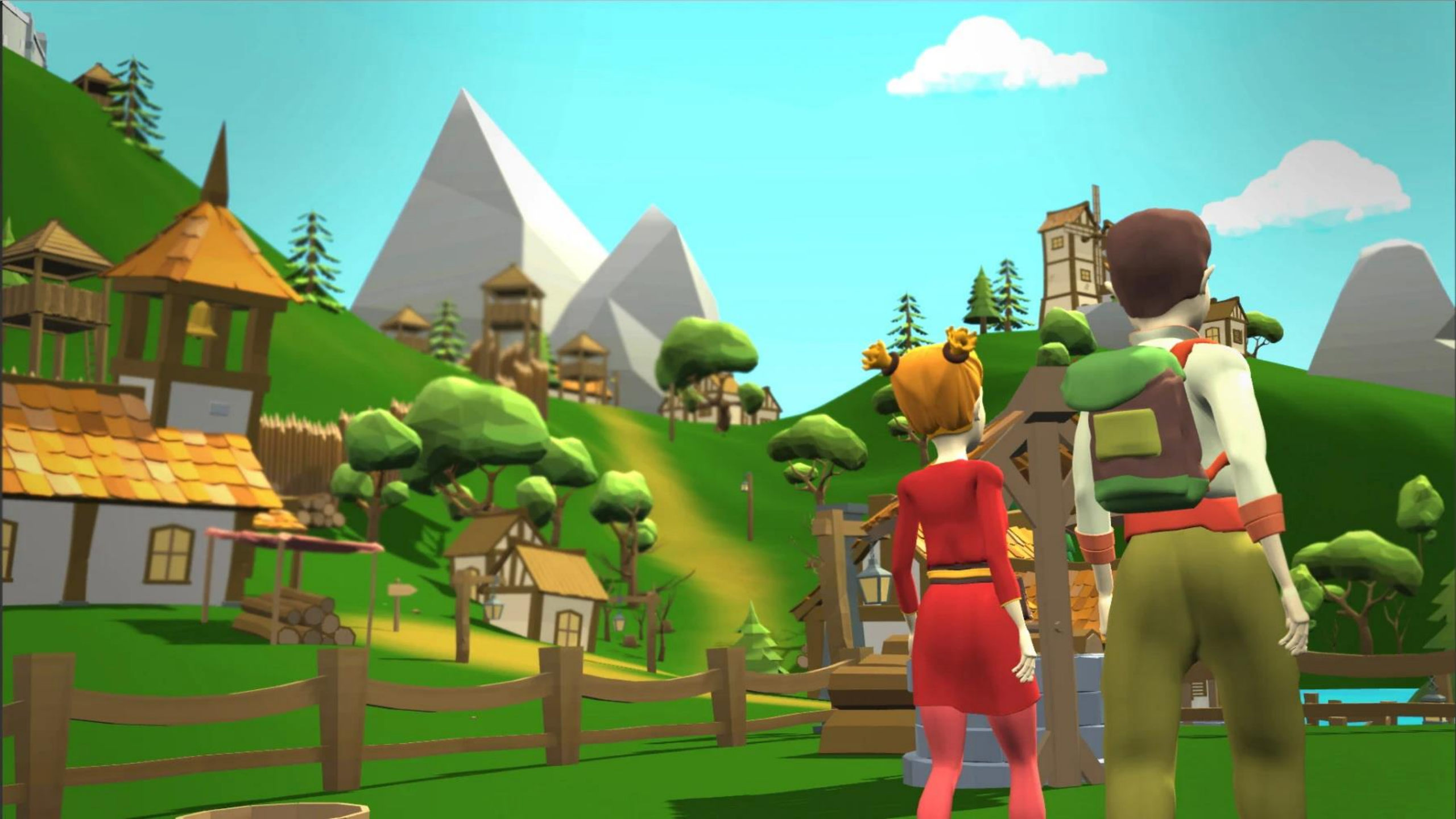

Supplement: Multimedia Appendix 1 [file formative_v8i1e53465_app1.pdf]
